# Supplementary material for: Uncultivated DPANN archaea are ubiquitous inhabitants of global oxygen-deficient zones with diverse metabolic potential
Source: mBio. 2024 Feb 21;15(3):e02918-23. doi: 10.1128/mbio.02918-23 (PMC10936187; doi:10.1128/mbio.02918-23)
Supplement: Supplemental Material — Supplemental text, Tables S1 and S2, and Figures S1-S3. [file mbio.02918-23-s0001.pdf]

Supplementary Material for

Uncultivated DPANN archaea are ubiquitous inhabitants of global oxygen-deficient zones with diverse metabolic potential

Irene H. Zhang[1#], Benedict Borer[1], Rui Zhao[1], Steven Wilbert[2], Dianne K. Newman[2,3], and Andrew R. Babbin[1#]

[1]Department of Earth, Atmospheric and Planetary Sciences, Massachusetts Institute of Technology, Cambridge, Massachusetts, USA

[2]Division of Biology and Biological Engineering, California Institute of Technology, Pasadena, California, USA

[3]Division of Geological and Planetary Sciences, California Institute of Technology, Pasadena, California, USA

[#]Address correspondence to Irene H. Zhang, izhang@mit.edu and Andrew R. Babbin, babbin@mit.edu

Table of Contents

Supplementary Methods

Supplementary References

Supplementary Tables S1–S2

Supplementary Figures S1–S3

Supplementary Methods

Sequence selection and genetic engineering

DPANN *nosZ*-like genes from ODZ metagenome-assembled genomes were extracted and aligned with MAFFT-linsi v7.450 using the --leavegappyregion parameters (1). Three representative sequences were obtained by clustering the DPANN *nos*-like genes at 90% nucleotide identity with vsearch (2). Putative *nosZ*-like gene sequences were optimized for the *Pseudomonas aeruginosa* genome using the IDT codon optimization tool. These fragments were synthesized as gBlocks from Twist Bioscience. Using gBlocks as template, the fragments were amplified with homologous overhangs using PCR primers (Table S1). Products were cloned into the pJM220 plasmid linearized by KpnI and HindIII digestion using Gibson assembly (NEB). Putative *nosZ*-like gene containing plasmids were introduced at the attTn7 site in the PA14(Δ*nosZ*) strain genome (3, 4). Strains with integrated plasmids will drive gene expression using a rhamnose-inducible promotor when grown in the presence of l-rhamnose.

Growth conditions and N_2O reduction tests

Three *Pseudomonas aeruginosa* PA14 Δ*nosZ* strains containing the putative *nosZ*-like genes were grown on LB plates at 37 °C overnight along with the parent wild-type PA14 (positive control) and PA14 Δ*nosZ* (negative control). Colonies were inoculated into 10 mL Luria-Bertani (LB) broth within serum vials. Serum vials containing each strain, along with a cell-free control, were capped and sealed post-inoculation and grown within a 37 °C incubator with shaking at 100 rpm. A total of 4 replicates were performed for each strain. After cultures used up the available oxygen to reach stationary growth phase, sterile 50 µmol L^{-1} nitrate and 0.02% l-rhamnose w/v were injected into each serum bottle to drive denitrification and the expression of the *nosZ*-like genes. After 24 hours of growth at 37 °C, N_2O concentrations were measured using a Unisense N_2O microelectrode connected to the Unisense Field Multimeter according to manufacturer protocols. N_2O concentrations were normalized to the average N_2O readout of the PA14 Δ*nosZ* control.

Supplementary References

1. Katoh K, Standley DM. 2013. MAFFT multiple sequence alignment software version 7: improvements in performance and usability. *Mol Biol Evol* 30:772–780.

2. Rognes T, Flouri T, Nichols B, Quince C, Mahé F. 2016. VSEARCH: a versatile open source tool for metagenomics. *PeerJ* 4:e2584.

3. Choi K-H, Schweizer HP. 2006. mini-Tn7 insertion in bacteria with single attTn7 sites: example *Pseudomonas aeruginosa*. *Nat Protoc* 1:153–161.

4. Jeske M, Altenbuchner J. 2010. The *Escherichia coli* rhamnose promoter rhaP(BAD) is in *Pseudomonas putida* KT2440 independent of Crp-cAMP activation. *Appl Microbiol Biotechnol* 85:1923–1933.

Supplementary Tables

	putnos1_DDOPOKPK_00765_Opt	putnos2_APMGHGFK_00118_Opt	putnos3_DAILHJJC_00524_Opt
Forward primer	CAGGAATTCCTCGAGAAGCTTATGAAGAATAAAGTCCTCATCATC	CAGGAATTCCTCGAGAAGCTTATGAAGAAGTACCTGCTCATC	CAGGAATTCCTCGAGAAGCTTATGCTCGTCGGGATCGTGTC
Reverse primer	GGGAACTGGTGGTCTCGTAAGGTACCTCGCGAATCAGAACG	CGTTCTGATTCGCGAGGTACCTTATTCGACCACGAGGGTCC	CGTTCTGATTCGCGAGGTACCTTACTTCACGATCAGCTTCCC

Table S1: Primers used to amplify DPANN *nosZ*-like gene fragments with overhangs for each of the 3 representative gene sequences.

**Parameter**	**Values**
Producer radius R [µm]	[0.5, 0.75, 1.0, 1.25, 1.5]
Consumer radius r [µm]	[0.05, 0.1, 0.25, 0.5]
Cell distance d [µm]	[0, 0.1, 0.5, 1, 2]

Table S2: List of parameters used in model including specific values used for all simulations.

Supplementary Figures

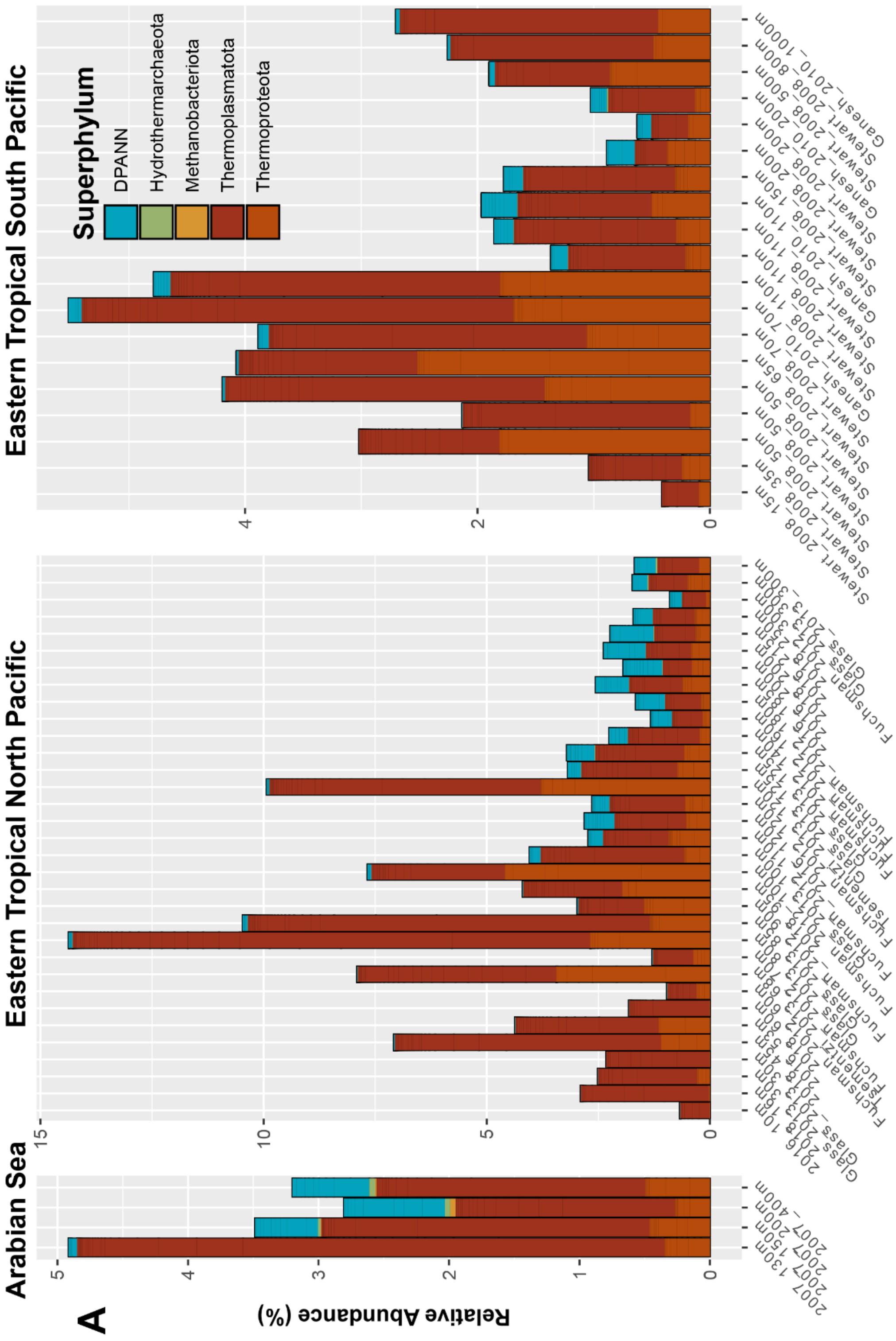

Figure S1 (continued on next page)

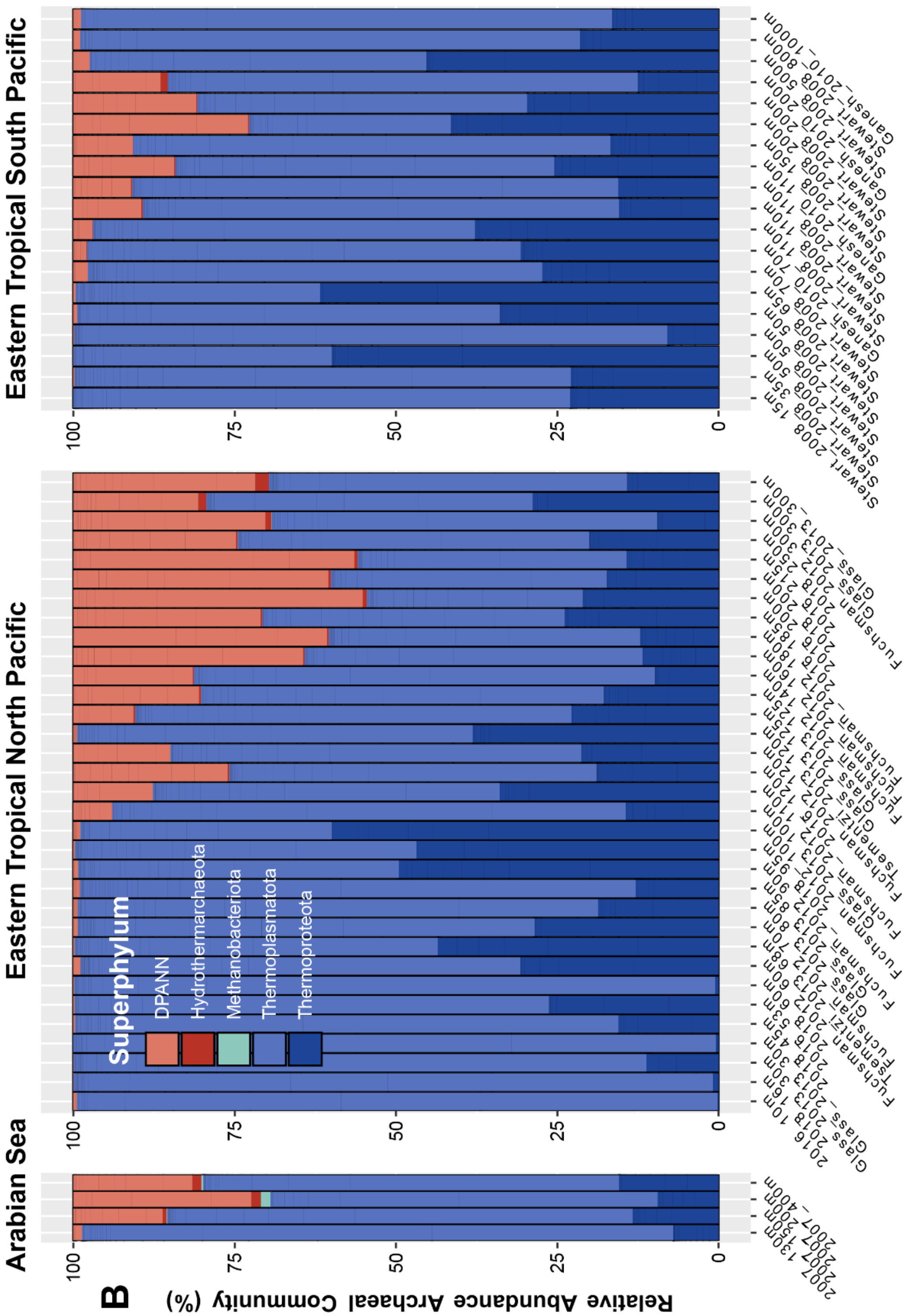

Figure S1: (A) Percentage of the total community assigned to the Archaea domain, with DPANN archaeal phyla grouped together (teal). Other archaea are colored by phylum. **(B)** Proportion of total archaeal reads belonging to the DPANN superphylum vs. other archaeal taxa, with DPANN archaeal phyla grouped together (orange). Other archaea are colored by phylum. All bars are scaled to 100%.

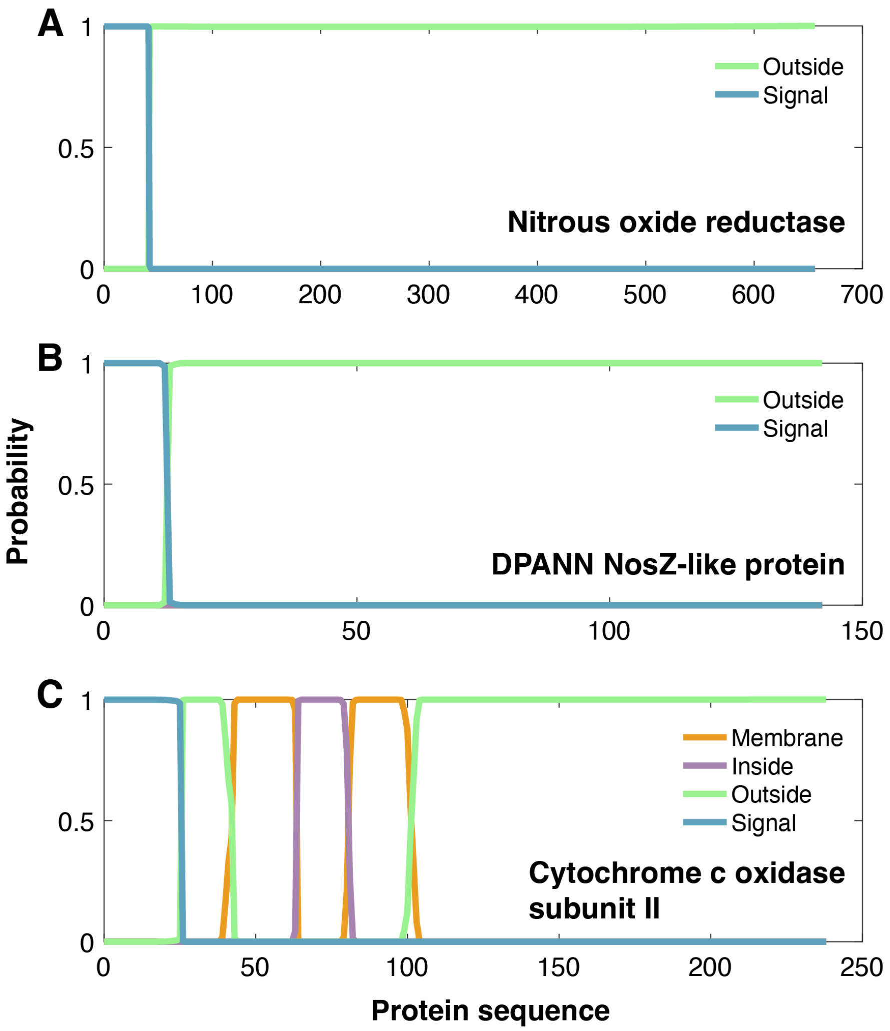

Figure S2: Prediction of protein location for **(A)** canonical *nosZ*-encoded protein **(B)** DPANN *nosZ*-like protein **(C)** cytochrome c oxidase subunit II. Protein topologies and locations were predicted using DeepTHHMM. Probability scores on the y-axis indicate predicted probability of the protein region as belonging to a signal peptide sequence (blue), cytoplasmic (inside membrane, purple), membrane-associated (orange), or outside the membrane such as in the periplasmic space (green). Amino acid positions are indicated on the x-axis.

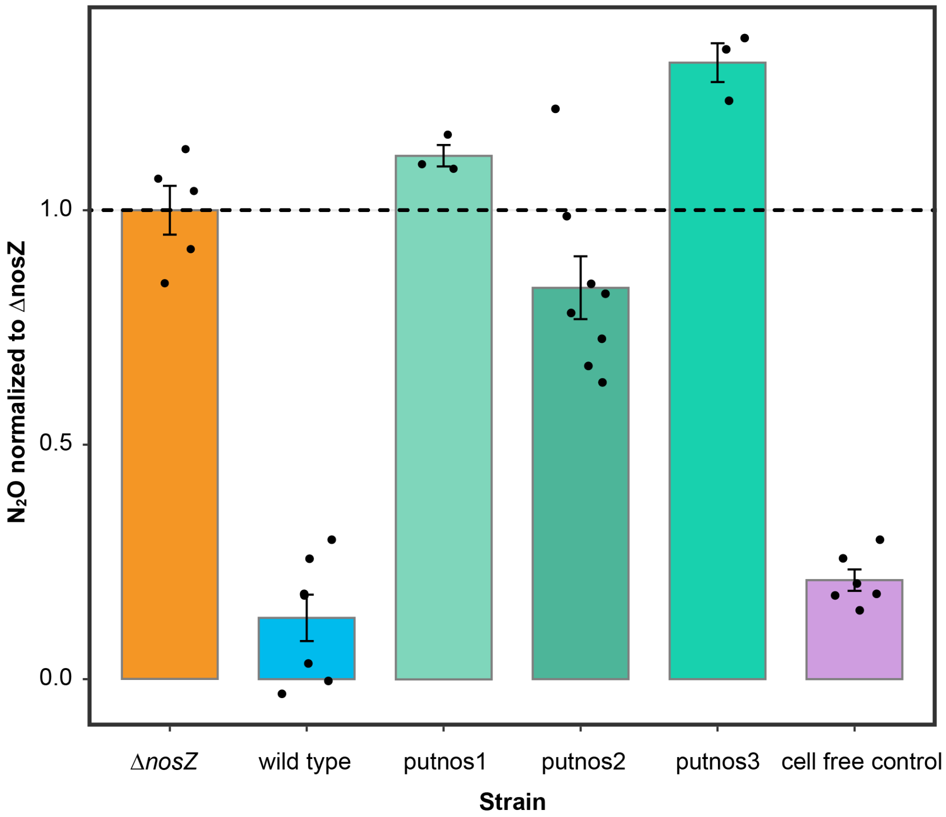

Figure S3: N_2O concentrations after 24 hours of anaerobic growth in LB supplemented with 50 µmol L^{-1} nitrate and 0.02% rhamnose to drive expression from the DPANN *nosZ*-like gene within the *P. aeruginosa* PA14 Δ*nosZ* background. N_2O concentrations are normalized to the average N_2O concentration for the parent PA14 Δ*nosZ* control (i.e., 25 µmol L^{-1} N_2O). The putnos1, putnos2, and putnos3 strains correspond to the 3 representative *nosZ*-like genes after clustering DPANN *nosZ*-like sequences at 90% nucleotide identity. Wild-type PA14 and a cell free control are also shown.
